# Observations of Inter‐Pack Conflict and Pack Instability in Contiguous Dingo Packs

**DOI:** 10.1002/ece3.74376

**Published:** 2026-09-26

**Authors:** Brendan F. Alting, Benjamin J. Pitcher, Michelle L. Campbell‐Ward, Neil R. Jordan

**Affiliations:** ^1^ Centre for Ecosystem Science, School of Biological, Earth and Environmental Sciences University of New South Wales (UNSW) Sydney Sydney New South Wales Australia; ^2^ Taronga Institute of Science and Learning Taronga Conservation Society Sydney New South Wales Australia; ^3^ School of Natural Sciences, Faculty of Science and Engineering Macquarie University Sydney New South Wales Australia; ^4^ Sydney School of Veterinary Science, Faculty of Science The University of Sydney Sydney New South Wales Australia

**Keywords:** animal movement, camera‐trap, canids, dingo, home‐range, pack conflict, territoriality

## Abstract

Using a combination of direct sightings, camera trap data, and GPS collar data, we report on dingo behaviour in two neighbouring packs in Myall Lakes National Park, Australia, following the death of two males from one pack due to inter‐pack conflict and (three weeks later) the death of the dominant male (one of the original aggressors) from an adjacent pack. The two packs occupied contiguous slightly overlapping home ranges prior to the inter‐pack killing. A period of social instability followed, with members of each pack detected beyond their original home ranges and forming new breeding pairs. Following the annual breeding season, and a period of approximately six weeks after the end of the conflict, territories had stabilised and approximated pre‐conflict territories. Understanding dingo territorial behaviour, particularly in response to the deaths of breeding individuals, may help to identify dingo responses to other forms of mortality, including lethal control which is a widespread management technique in Australia, for which the social consequences are debated.

## Graphical abstract

We observed the behavior of dingoes before, during and after dominant dingoes from adjacent packs were killed in presumed intraspecific conflict. Dingo territorial borders of the two packs changed, with altered associations between individuals of different packs. Territorial borders re‐solidified after six weeks, with some individuals switching packs.

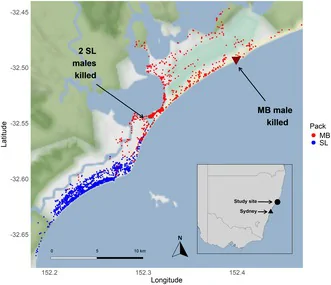

## 1 Introduction

In some canids, ranging increases prior to and during the breeding season, and conflicts between adjacent packs can occur (Gese 2001). Inter‐pack conflicts can be difficult to observe however, particularly for wide‐ranging and cryptic species (Jordan et al. 2017), and anecdotal observations can assist in the accumulation of meaningful data (Cassidy et al. 2015). Dingoes (*Canis familiaris, Canis dingo, Canis lupus dingo* amongst others, IUCN status LC; Kreplins et al. 2018) are a territorial social canid, with family groups or ‘packs’ typically centred around a socially dominant breeding pair, which breed annually from March to June (Jones and Stevens 1988). The rest of the pack consists of some offspring born in previous years that remain in the natal pack for a period of time and may assist in rearing additional litters in subsequent years, potentially also having litters of their own (Tatler et al. 2021). In undisturbed populations, most reported dingo territories are clearly defined, with limited spatial overlap between packs (Thomson 1992).

How territoriality and social structures manifest during the breeding season is unclear for dingoes, as interactions (including aggression) between packs are rarely witnessed directly (Thomson 1992). Behavioural responses of dingoes to resultant changes in social hierarchies are thus also unclear. This has flow‐on effects for dingo management, as the social consequences of management actions taken to reduce the negative impacts of dingoes in various contexts are uncertain. In some carnivores (e.g., grey wolves *Canis lupus*), the death of a socially dominant individual precedes social dissolution and subsequent reorganisation in packs (Borg et al. 2015). Given the broad scale lethal control of dingoes across much of Australia, insights into the behavioural responses of packs to the deaths of dominant individuals are valuable in assessing and predicting population responses to management actions.

**FIGURE 1:**
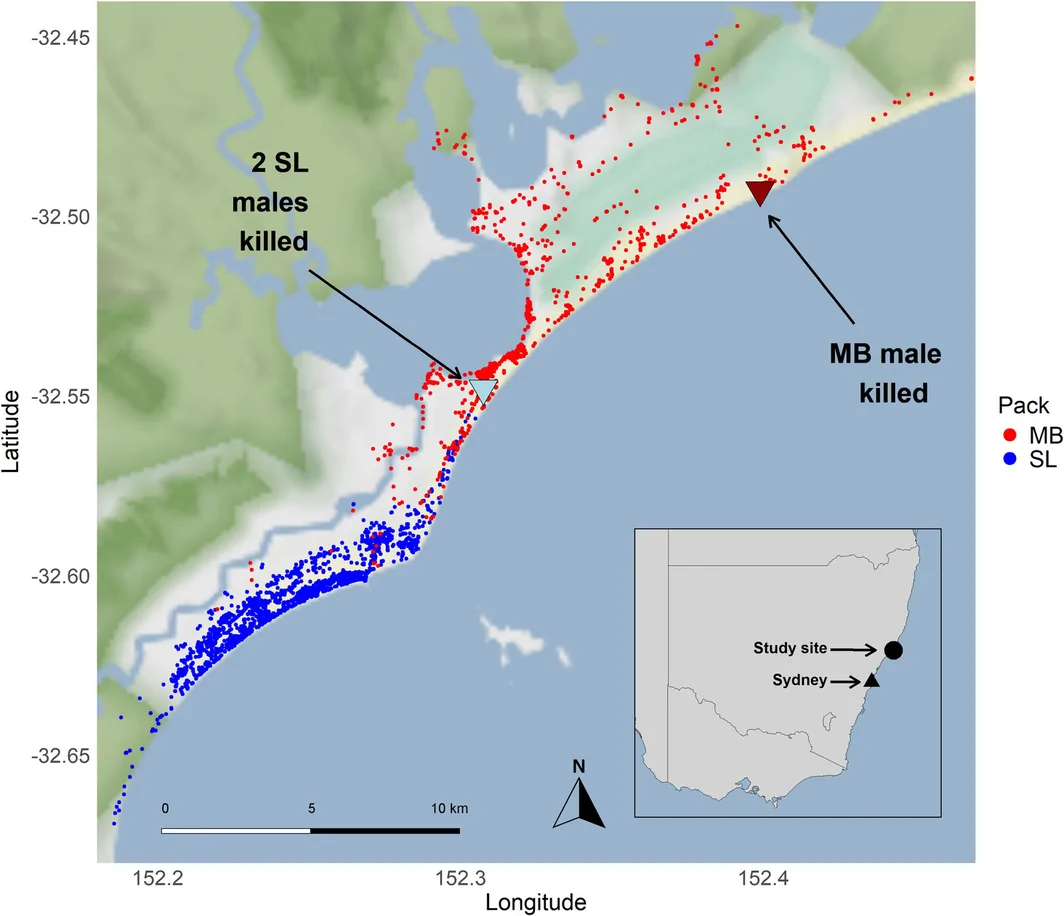
GPS collar data representative of two dingo packs (named SL and MB) leading up to the deaths of two males from SL pack. MB male was killed one month later. Dots represent hourly locations, colour‐coded by pack. Study site on the mid‐coast of NSW, Australia. Data from 2022, figures made using ggplot2 (Wickham 2016), rnaturalearth (Massicotte and South 2025), and ggmap (Kahle and Wickham 2013).

Here we describe dingo movements that occurred after three adult male dingoes (one dominant, two subdominants) from one pack (MB Pack) killed two adult males (one dominant, and one subdominant), from a neighbouring pack (SL Pack). We also report a third killing a month later, this time of the dominant male dingo involved in the initial killing. All dingoes were individually identifiable and were known members of packs, with known ranges (Figure 1) and compositions (Table 1). The initial intraspecific killing event was witnessed and filmed by local land managers, and we utilise data from a camera trap array deployed prior to and running beyond the inter‐pack conflict to identify any broad changes in movement and space use for each individual dingo. We also observed and report scent marking behaviours from these packs to illustrate dominance changes, and report reproductive events witnessed by local managers. Given the prevalence of lethal control of dingoes in Australia, how dingo packs respond to the death of pack members is of interest but not well understood.

**TABLE 1:** Summary of pack structures for two adjacent dingo packs in the Myall Lakes region from the mid‐coast of NSW, Australia, both before and after a conflict between the packs and the deaths of three dingoes.

Pack	Status	Pre conflict pack members	Post conflict pack members
SL	Dominant	SLF1501, PCM1601—	SLF1501, UOM2002+
	Subdominant	SLM2005‐, SLF2001*, SLF2003*	None
MB	Dominant	UOF1801, UOM1701—	UOF1801, UOM2001+
	Subdominant	UOM2001, UOM2002+	None

*Note:* ‐ = killed in conflict. + = immigrated/emigrated and assumed dominance in new pack. * = dispersed/not seen again.

## 2 Methods

Observations were made on Worimi Country in the Great Lakes region of the mid‐coast of Eastern Australia (centred on 32.492° S, 152.343° E). Dingo pack ranges were approximated from hourly location data collected on GPS collars fitted to a resident individual in both neighbouring dingo packs, a reliable method of pack home range estimation for canids (Benson and Patterson 2015). Two dingoes were collared in April 2021, and two in April 2022. To fit these collars (Advanced Telemetry Systems W500), dingoes were trapped in large walk‐in cages baited with fish or kangaroo meat and anaesthetised by an experienced wildlife veterinarian employing an intramuscular injection of a combination of medetomidine (0.04–0.05 mg/kg) and ketamine (3.1–4.0 mg/kg) via a blow dart. This was reversed following collaring with an intramuscular injection of atipamezole (5 × the medetomidine dose).

GPS collar data were downloaded via a UHF link during monthly welfare checks and processed in R (R Core Team 2025). Approximate dingo pack home ranges were determined by applying 95% minimum convex polygons to GPS fixes using the adehabitatHR package (Calenge 2024). Fixes from individuals collared in 2021 were used to estimate pre‐conflict home ranges (December 2021–March 2022), while fixes from individuals collared in 2022 were used to estimate post‐conflict pack home ranges (June 2022–September 2022). GPS fixes used in analysis did not overlap the study periods of ‘before’, ‘during’, and ‘after’ outlined below. Packs in the area have been under study since 2019, and all individuals within the study area were previously known to researchers (Alting et al. 2024, 2025). On‐ground observations from researchers and local managers also corroborated dingo pack structures leading up to the inter‐pack deaths.

Twenty‐two trail camera stations (two cameras per station, Reconyx Hyperfire H2 × 1000, minus one stolen) were deployed as part of a broader dingo population survey from December 2021–June 2022 (Alting et al. 2024). Camera stations were positioned ~2500 m apart, one on either side of a trail or road facing inwards, secured to trees approximately ~50 cm off the ground to capture both flanks of passing dingoes. Cameras were set to take 3 images per burst, with no trigger delay and set to medium‐high sensitivity. Image processing and dingo identification was done according to methods laid out in Alting et al. (2024), with dingoes identified using their unique sock patterns and other distinctive marks. All nighttime images and any daytime images where individuals could not be identified were discarded. Dingoes from these two packs were detected at 14 of the 22 camera stations.

We defined the period “before” the pack disruption as 12/02/22–26/03/2022 (43 days), the period “during” the conflict as 27/03/2022–08/05/2022 (43 days), and “after” 09/05/2022–31/05/2022 (22 days), as the camera survey ended at this point. The start and end dates of the ‘during’ period are when the two inter‐pack killing incidents occurred. For each dingo from each pack, we recorded whether they were detected at cameras within their pack's estimated range, or outside it, for each of the three time periods, and then calculated the proportion of detections within each of these zones. We analysed only proportions of detections within each zone, as raw counts would be unsuitable given the deaths of some dingoes during the study period (meaning they could no longer be captured on camera), and given ‘before’ and ‘during’ periods were longer than the ‘after’ period. All data processing and analysis were conducted in R (R Core Team 2025).

Additional cameras (Browning strike force HD pro) were deployed at specific scent marking sites within the region for a separate study (Alting et al. 2025), with one site within the ranges of the study packs. Cameras were positioned high in trees to avoid theft in conspicuous areas, primarily junctions of trails and roads. Cameras were set to record videos for 30s, with no trigger delay and set to high sensitivity. Videos were processed manually by one researcher, and individual dingo detections and scent marking behaviours including urinations, ground rakes, defecations, and overmarking (urinating over a scent mark of another individual) were recorded in a spreadsheet. Here we report observations of behaviours from identified dingoes at this site.

Opportunistic observations of copulations and sightings of individuals, pairs, and groups of dingoes were also recorded by researchers and on‐ground managers familiar with the individual dingoes. Individual identification was confirmed by researchers using images and video footage with reference to an established database.

## 3 Results

The structure and demographic composition of the two core study packs‐ the Mungo Brush pack (MB) and the Stewart and Lloyds pack (SL)– are outlined in Table 1. Three of nine individuals in the two packs were of known origin and age, while others were adults at the onset of the broader study in 2019 or immigrated from outside the study population since then. UOM2001 and UOM2002 had been seen as juveniles with the dominant adults in the MB pack, but parentage was never confirmed and these individuals may have been adopted from another litter (MLDP unpublished data). The three subdominant individuals in the SL pack were all confirmed pups of the dominant female SLF1501, and presumed sired by the dominant male PCM1601. The two collared dingoes from each pack were UOM2002 for the MB pack and SLF2003 for SL pack.

### 3.1 Start of Conflict Period‐ Death of 1 Dominant and 1 Subdominant Male From SL Pack

On 27/03/2022, three male dingoes from the MB pack: UOM1701 (dominant), UOM2001 (subdominant), UOM2002 (subdominant) were observed killing the dominant male of the SL pack, PCM1601. A subdominant male SLM2005, also from SL pack, was later found dead nearby and assumed killed in the same incident. The conflict occurred on a main road at the interface of these two packs' territories (Figure 1), just inside the 95% range of the MB pack. While cannibalism has been demonstrated in dingoes in a desert environment (Meek and Brown 2016), both were left in situ and uneaten by the MB pack in this instance.

### 3.2 2nd Conflict and Death of Dominant Dingo From MB Pack

On 09/05/2022, the research team received reports of a dead dingo on the beach within the MB pack's pre‐conflict range (Figure 1), with bite wounds to the neck, consistent with a dingo attack (Behrendorff et al. 2018). This was confirmed to be individual UOM1701, although which dingo or dingoes killed this male is unclear as the event was unobserved.

After this death, the packs stabilised socially. UOM2002 and SLF1501 formed the dominant pair in the SL pack, and UOM2001 and UOF1801 formed the dominant pair in the MB pack. At the time of writing (August 2026, four years later), both pairs remain dominant in their packs. GPS collar data from relevant individuals in each pack (UOF1801 in MB, and SLF1501 in SL) from June–September 2022 showed that both packs reverted to their pre‐conflict home ranges (Figure S1). The home ranges of these two packs have subsequently remained stable to August 2026.

### 3.3 Camera Trap Detections

**FIGURE 2:**
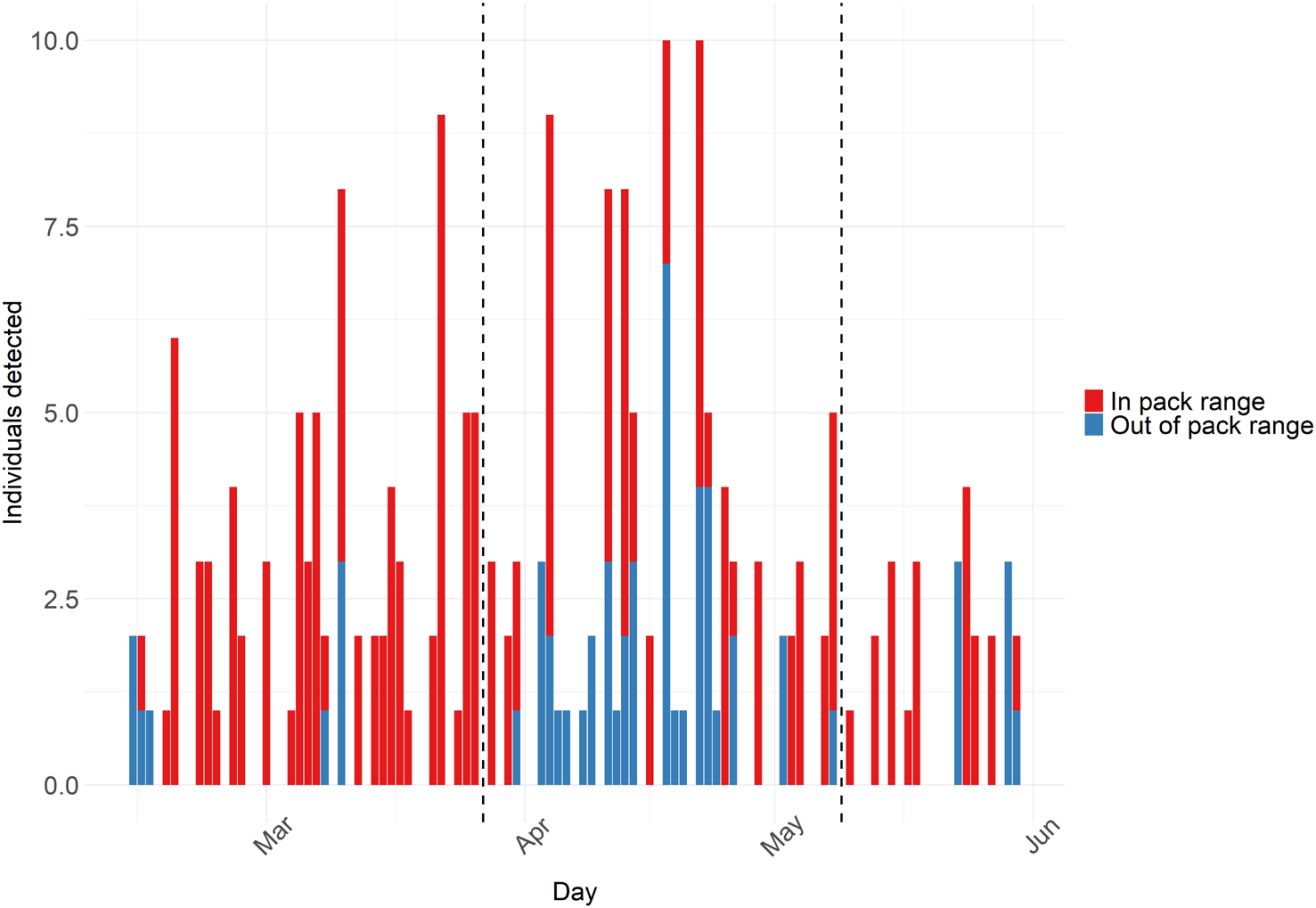
Combined detections of nine individual dingoes (five SL pack, four MB pack) summed by day, with detections grouped as either inside or outside their home range. First dotted line is the death of two males from SL pack; second dotted line is death of dominant male from MB pack. Camera survey ended 31/05/2022. Figures made in R (R Core team 2025) with ggplot2 (Wickham 2016).

We recorded 215 independent dingo detections across the relevant timeframe (Table 2; Table S1). When controlling for the number of dingoes alive during each period, daily detections of dingoes were higher before the conflict period than during or after (Table 2; Figure 2). Dingoes were detected proportionally more outside their home range during the conflict period than before or after (Table 2). From 27/03/2022 onwards, the dominant female from SL pack was seen with males from MB pack eight times, six times with two MB pack males simultaneously. Dominance status amongst the males was unclear for this period, and all three adult males from MB pack were seen on camera traps with the dominant females of both packs (22 times) on separate occasions over a four‐week period. These two females were never seen together on cameras and seemed to retain spatial segregation between each other. The males however appeared to be in flux, alternating associations with the two females over the territorial re‐establishment period. For example, UOM2002 from MB pack was detected with the dominant female from SL pack once, and with the dominant female from MB pack fifteen times in the six weeks after the death of the SL males, but has subsequently not been detected with the MB pack dominant female (as of August 2026).

**TABLE 2:** Summary statistics and proportions of total dingo detections from two packs inside and outside their home range for each time period (before conflict, during conflict, after conflict).

Period	Detections within the home range (per day)		Detections outside the home range (per day)		Proportion of detections in home range (%)	Proportion of detections outside home range (%)
	Total	Per alive dingo	Total	Per alive dingo		
Before conflict period (*n* = 10 dingoes alive)	80 (1.86)	8 (0.186)	8 (0.186)	0.8 (0.019)	90.9	9.1
During conflict period (*n* = 8 dingoes alive)	58 (1.349)	7.25 (0.169)	43 (1)	5.375 (0.125)	57.4	42.6
After conflict period (*n* = 7 dingoes alive)	19 (0.864)	2.714 (0.123)	7 (0.318)	1 (0.045)	73.1	26.9

*Note:* Data from camera trap surveys conducted in Myall Lakes National Park in 2022.

### 3.4 Scent Marking During Conflict Period

On 18/04/2022, we recorded the SL pack dominant female scent marking at a marking site, with two MB pack males (UOM2001 and UOM1701) also present. UOM2001 appeared as if interested in overmarking SLF1501's scent mark but was followed closely and seemingly aggressively by UOM1701. UOM2001 did not overmark the female, and UOM1701 finally overmarked the initial female scent instead.

### 3.5 Direct Observations of Copulations

**FIGURE 3:**
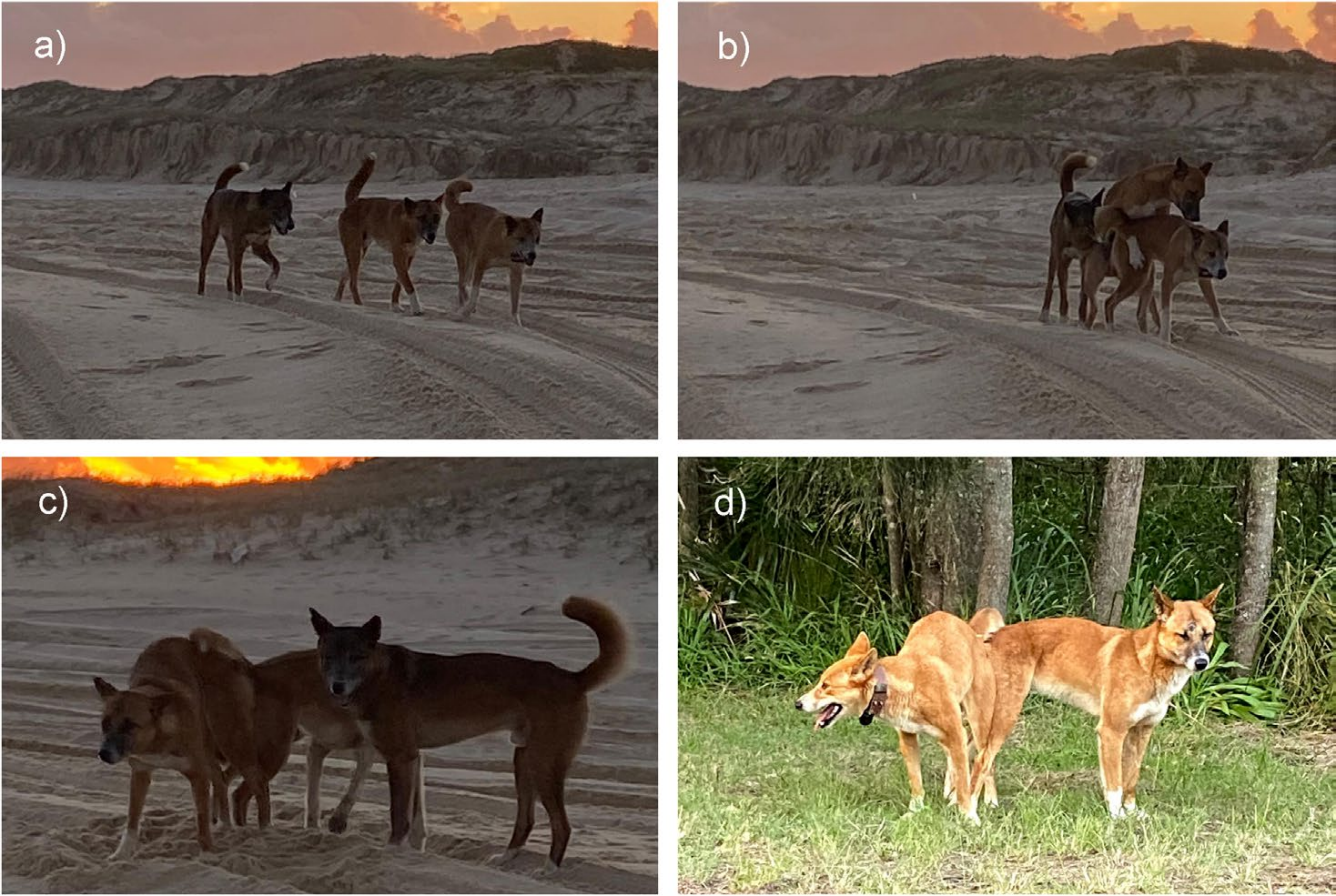
Instances of dingoes breeding during a period of territorial re‐adjustment post inter‐pack conflict. 21/04/2022: UOM2001 (previously subdominant in MB pack) was filmed breeding with SLF1501 (dominant in SL pack) (a–c) while UOM1701 (previously dominant in MB pack) is present in SL territory. 19/05/2022: UOM2001 breeding with UOF1801 (dominant in MB pack) (d) in MB territory. Photo credit Nick Patteson.

Each of UOM2001, UOM2002 and UOM1701 was observed mating with the female SLF1501 during the period of territorial re‐establishment. On 21/04/2022, UOM2001 (previously subdominant) was observed breeding with SLF1501 while UOM1701 (previously dominant) was present, indicating a shift of dominance (Figure 3a–c). SLF1501 had a litter of pups that year, although the paternity of this litter is unknown. UOM2002 helped to rear these pups. SLF1501 also had litters in 2023,2024 and 2025. On 19/05/2022, UOM2001 was also pictured breeding with UOF1801 (Figure 3d), although she did not have a litter that year or indeed in any subsequent years (four breeding seasons).

## 4 Discussion

Here we have described altered ranging behaviours and scent marking strategies of dingoes which indicated shifting dominance statuses between individuals within and between dingo packs. The results suggest that periods of re‐alignment in canid populations may occur post death of dominant individuals, during which behaviours of remaining individuals in the area may become unpredictable.

### 4.1 Evidence for Intraspecific Competition and the Consequences for Dominants

Given the location of the killing of UOM1701 (within the MB home range), and the lack of observations from either camera traps or on ground observations of other dingoes in the area at that time, it seems most probable that UOM1701 was killed by one of either UOM2001 or UOM2002, his previous packmates and subordinates, as both lived after this point and became dominant in two different packs. We also note aggressive encounters observed at a scent marking site between UOM2001 and UOM1701 (discussed below). Despite this, there is no way to definitively confirm by which individual UOM1701 was killed. Further evidence that these conflicts were the result of intraspecific conflict is that carcasses of SL individuals from the initial deaths were not consumed by the MB pack. This aligns with similar findings from K'gari (Behrendorff et al. 2018) of intraspecific conflict, although other authors report incidences of cannibalism of dingoes killed for lethal control (Meek and Brown 2016; Smith et al. 2020).

Multiple dingoes were observed mating with SLF1501, and that UOM2002 helped raise the subsequent litter from that year perhaps highlights a benefit of confusing paternity for female dingoes (Macdonald et al. 2019). Despite this, UOM2002 is assumed to be the father of three subsequent litters raised in the SL territory (2023, 2024, 2025), as no other males have been seen with SLF1501 since. The potential costs of rearing a litter that did not definitely contain his (UOM2002) offspring in 2022 were perhaps negated by the potential to successfully raise litters in subsequent years, as a direct result of attaining dominance status in the SL pack. Additional individuals within a pack may also benefit territory holders if there are sufficient resources to support them, as larger packs generally win in conflicts between packs (Cassidy et al. 2015).

### 4.2 Scent Marking Behaviours

The three male dingoes from the pre‐conflict MB pack displayed unusual scent marking patterns during the period of conflict that were inconsistent with those of established packs with a clear dominant male, as occurs in other canids (e.g., African wild dogs *(Lycaon pictus)*, Jordan et al. 2014). UOM2001 appeared interested in overmarking SLF1501, which is a typical display of pair bonding between individuals (Jordan et al. 2014). UOM1701's constantly vigilant behaviour around UOM2001 during this event was unusual for a dominant dingo against a subdominant, particularly one raised by the dominant from juvenile age, and may be suggestive of a power struggle between these two individuals. Male dingoes have been shown to increase scent marking during the breeding season (Alting et al. 2025), and it is plausible that the breeding season may be a trigger period for pack takeovers by subordinates, as well as increasing forays by subordinates into adjacent territories, as occurs in other species (Young et al. 2007). This may come with a greater risk of intraspecific conflict for territory holders during the breeding period.

### 4.3 Female Ranging Patterns

Ranges of adjacent packs post‐conflict approximated the borders of pack home ranges pre‐conflict. That both dominant females remained in their existing ranges, whilst males moved, hints that females may be contributing to home range stability and extent more than males are (Johansson et al. 2018). This has been hypothesised and described in other territorial systems, in which female territories are centred around resource availability, and male territories around females (Lukas and Clutton‐Brock 2013). The phenomenon of females contributing disproportionately to their pack's territorial extent in dingoes is understudied and requires further research.

## 5 Conclusions

Our observations align with the literature around the structuring of social systems of territorial species when dominant individuals die. In a study of red foxes ( *Vulpes vulpes* ), the death of a dominant male preceded considerable social disruption within the vacant territory in which many more subordinate individuals were sighted in the area than when the dominant controlled the territory (Dorning and Harris 2019). In a study of coyotes, upon the death of one dominant male and abandonment of their territory by the dominant female, a stable adjacent coyote pack shifted its territory partially into the then vacant territory (Gese 1998). Increases in subordinate activity and territorial expansion by adjacent packs as seen in these two studies are consistent with what we observed in this system and suggest that social disruption, particularly during the breeding season, provides opportunities for other individuals.

That dingo ranges appeared to be in flux during the period of conflict has implications for our understanding of ranging behaviour under different management actions. Indiscriminate lethal control of dingoes through poison baiting is practiced across Australia, and the deaths of dominant individuals from packs may cause or influence social instability (Wallach et al. 2009). This may make ranging patterns of dingoes less predictable, potentially resulting in undesirable management outcomes. The observed patterns of behaviour and territorial re‐alignment may not be representative of all dingo packs following the death of dominant individuals, but do provide valuable insights into a likely common but little studied phenomenon. The process may differ, for example, where dominant females are killed. Understanding how pack dominance status and territorial establishment in dingoes' manifests requires further study.

## Author Contributions

**Brendan F. Alting:** conceptualization (equal), data curation (lead), investigation (equal), writing – original draft (lead), writing – review and editing (lead). **Benjamin J. Pitcher:** conceptualization (equal), investigation (equal), writing – original draft (supporting), writing – review and editing (supporting). **Michelle L. Campbell‐Ward:** investigation (equal), writing – original draft (supporting), writing – review and editing (supporting). **Neil R. Jordan:** conceptualization (equal), investigation (equal), writing – original draft (supporting), writing – review and editing (supporting).

## Funding

This work was supported by Taronga Conservation Society Australia.

## Conflicts of Interest

The authors declare no conflicts of interest.

## Supporting information


**Figure S1:** Minimum convex polygons (95%) constructed around hourly GPS fixes from four representative dingoes from two different packs. (a) Represents four months of data directly preceding a period of inter‐pack conflict (December 2021–March 2022). (b) Represents three months of data directly after the period of conflict (June–September 2022).
**Table S1:** Total detections of individual dingoes summed across all camera traps per individual for each conflict period. Data from a study in Myall Lakes National Park during a period of interpack conflict between adjacent dingo packs, March–May 2022.

## Data Availability

Data used in this analysis has been deposited to dryad: https://doi.org/10.5061/dryad.hqbzkh1zx.
