# Supplementary material for: Observations of Inter‐Pack Conflict and Pack Instability in Contiguous Dingo Packs
Source: Ecol Evol. 2026 Sep 26;16(9):e74376. doi: 10.1002/ece3.74376 (PMC13615896; doi:10.1002/ece3.74376)
Supplement: Supplementary file 1 [file ECE3-16-e74376-s001.docx]

Supplementary materials:

Supplementary figure 1: Minimum convex polygons (95%) constructed around hourly GPS fixes from four representative dingoes from two different packs. a) represents four months of data directly preceding a period of inter-pack conflict (December 2021-March 2022). b) represents three months of data directly after the period of conflict (June-September 2022).

Supplementary table 1: Total detections of individual dingoes summed across all camera traps per individual for each conflict period. Data from a study in Myall Lakes National Park during a period of interpack conflict between adjacent dingo packs, March-May 2022.

| Individual | Pack | Detections Before Conflict | Detections During Conflict | Detections After Conflict | Fate |
| --- | --- | --- | --- | --- | --- |
| PCM1601 | SL | 7 | 0 | 0 | Killed |
| SLF1501 | SL | 7 | 14 | 2 | Retained Dominance |
| SLF2001 | SL | 5 | 0 | 0 | Dispersed/Disappeared |
| SLF2003 | SL | 4 | 5 | 0 | Dispersed/Disappeared |
| SLM2005 | SL | 8 | 0 | 0 | Killed |
| UOF1801 | MB | 13 | 18 | 9 | Retained Dominant |
| UOM1701 | MB | 23 | 27 | 0 | Killed |
| UOM2001 | MB | 4 | 17 | 10 | Newly Dominant |
| UOM2002 | MB-> SL | 17 | 20 | 5 | Newly Dominant |
|  |  | **88** | **101** | **26** |  |
